# “A rising tide lifts all boats”: establishing a multidisciplinary genomic tumor board for breast cancer patients with advanced disease

**DOI:** 10.1186/s12920-016-0234-1

**Published:** 2016-11-21

**Authors:** Michelle L. McGowan, Roselle S. Ponsaran, Paula Silverman, Lyndsay N. Harris, Patricia A. Marshall

**Affiliations:** 1 Ethics Center, Division of General and Community Pediatrics, Cincinnati Children’s Hospital Medical Center, Departments of Pediatrics and Women’s, Gender, and Sexuality Studies, University of Cincinnati, 3333 Burnet Avenue, MLC 15006, Cincinnati, OH 45229 USA; 2 Case Western Reserve University School of Medicine, Cleveland, USA; 3 Case Western Reserve University School of Medicine, University Hospitals Seidman Cancer Center, Cleveland, USA; 4 National Cancer Institute, Washington, DC USA

**Keywords:** Breast cancer, Genomics, Qualitative research, Patient care, Ethics

## Abstract

**Background:**

Research suggests that multidisciplinary genomic tumor boards (MGTB) can inform cancer patient care, though little is known about factors influencing how MGTBs interpret genomic test results, make recommendations, and perceive the utility of this approach. This study’s objective was to observe, describe, and assess the establishment of the Breast Multidisciplinary Genomic Tumor Board, the first MGTB focused on interpreting genomic test results for breast cancer patients with advanced disease.

**Methods:**

We conducted a qualitative case study involving participant observation at monthly MGTB meetings from October 2013 through November 2014 and interviews with 12 MGTB members. We analyzed social dynamics and interactions within the MGTB regarding interpretation of genomic findings and participants’ views on effectiveness of the MGTB in using genomics to inform patient care.

**Results:**

Twenty-two physicians, physician-scientists, basic scientists, bioethicists, and allied care professionals comprised the MGTB. The MGTB reviewed FoundationOne™ results for 40 metastatic breast cancer patients. Based on findings, the board mostly recommended referring patients to clinical trials (34) and medical genetics (15), and Food and Drug Administration-approved (FDA) breast cancer therapies (13). Though multidisciplinary, recommendations were driven by medical oncologists. Interviewees described providing more precise care recommendations and professional development as advantages and the limited actionability of genomic test results as a challenge for the MGTB.

**Conclusions:**

Findings suggest both feasibility and desirability of pooling professional expertise in genomically-guided breast cancer care and challenges to institutionalizing a Breast MGTB, specifically in promoting interdisciplinary contributions and managing limited actionability of genomic test results for patients with advanced disease.

## Background

Enthusiasm is increasing among oncologists for using molecular tests to guide the application of targeted cancer therapies [1–3]. However, despite the rapid integration of genomic tests [4], care paths for using test results to guide treatment do not yet exist. Given concerns regarding insurance coverage and cost effectiveness of genomic testing and uncertain ethical implications of genomic information, establishing protocols for administering and delivering genomic risk information to patients is important [2, 5, 6].

Previous studies suggest that interdisciplinary groups of experts can inform genomically-guided patient care [7–9]. Cancer centers are establishing sequencing tumor boards or multidisciplinary genomic tumor boards (MGTBs) to review tumor sequencing results and identify potential therapies for patients [10–14]. MGTBs are modeled after widely-practiced, disease-specific tumor boards comprised of oncology, radiology, and pathology specialists who pool their expertise to review newly-diagnosed and challenging patients’ cases [15]. Research suggests that multidisciplinary cancer care teams can improve patients’ therapy planning, pain control, and medication adherence, though findings supporting their effectiveness in impacting patient survival and the costs of care are limited [16–18]. This may be due in part to discordance between recommendations and patient values and lack of follow up on tumor board recommendations [19, 20]. Nevertheless, studies suggest that multidisciplinary tumor boards and the use of standardized templates in tumor board conferences can improve oncology patient outcomes based on proxy measures such as the adherence to standards of care and national guidelines for treatment [21, 22].

At this time little is known about the similarities and differences in how traditional multidisciplinary tumor boards and MGTBs impact patient care. What is known is that an MGTB diverges from typical multidisciplinary tumor boards by including professionals with expertise in clinical or basic sciences relevant to genetics and genomics, bioinformatics, and bioethics. For instance, The Michigan Oncology Sequencing Project utilized a mock MGTB to assess the clinical feasibility of implementing tumor sequencing to identify patients for biomarker-driven clinical trials [10]. Investigators at The Moores Cancer Center reported that their molecular tumor board involved participants from medical oncology, medical genetics, pathology, bioinformatics, and basic and translational science who analyzed patients with cancer diagnoses who had, on average, three prior therapies [13]. They argued that for a heavily pretreated population of patients with advanced disease, genomic testing and a molecular tumor board’s recommendations could optimize patient management, though limited access to targeted drugs and clinical trials pose a hindrance [15]. Miller and colleagues found that physicians were generally optimistic about the long-term potential for genomic tumor analyses for metastatic cancer patients, but more conservative about short-range benefits for patients undergoing genomic sequencing today [14].

Limited information is available on factors influencing how MGTBs interpret test results and make patient care recommendations and members’ attitudes towards the utility of this approach in guiding patient care. We report on a qualitative study to observe, describe, and assess the establishment of the Breast Multidisciplinary Genomic Tumor Board, the first MGTB specifically focused on interpreting genomic test results for breast cancer patients with advanced disease.

## Methods

We conducted a 1-year qualitative case study employing participant observation and in-depth interviews to study the MGTB and its participants. The study was approved by the University Hospitals Case Medical Center Institutional Review Board. All individuals participating in MGTB meetings in-person and via phone- or web-conference signed informed consent documents indicating their voluntary participation in the study. Participant observation was implemented at monthly meetings of the MGTB from October 2013 through November 2014. Participant observation refers to an approach in which researchers are embedded in a social environment and engaged in its ongoing activities to understand interactions between individuals and meanings attached to experiences and behavior [23, 24]. McGowan and Ponsaran took field notes describing observations of each meeting, providing us with an opportunity to observe the evolution of the practices of the MGTB throughout the first year that it met. All participants were invited to participate in an interview about their involvement with the MGTB. After the initial round of volunteers was secured, we sought breadth in professional backgrounds to reduce the potential for bias in the sample and to achieve theoretical saturation [25]. The interview guide can be found in the Appendix. Meeting proceedings and interviews were audio-recorded for transcription and data analysis, which involved thematic analysis of qualitative text data and field notes [25]. We analyzed social dynamics and interactions within the MGTB regarding interpretation and communication of genomic findings and participants’ views on effectiveness of the MGTB approach to incorporating genomic findings into patient care, organizing the data into the themes of MGTB practices, perceived benefits of the MGTB, and perceived challenges of the MGTB.

## Results

**Fig. 1:**
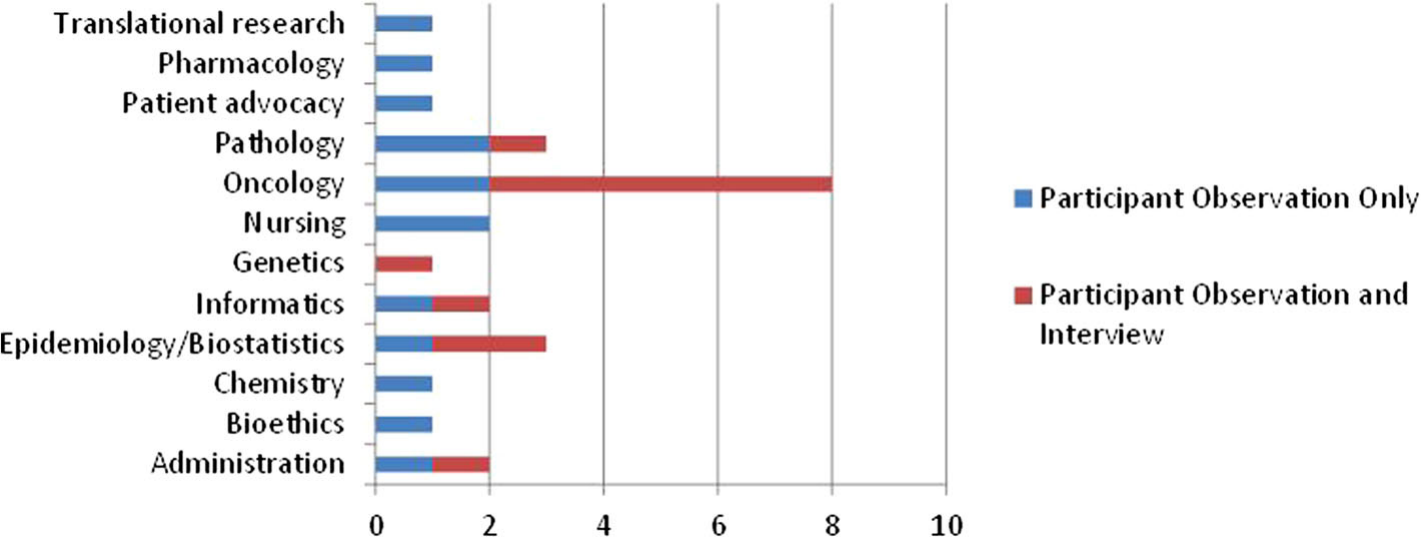
MGTB participants by specialty

The MGTB was launched in October 2013 and met for 60–90 min each month. The MGTB was comprised of 26 individuals representing medical, surgical, and radiation oncology, pathology, genetics, epidemiology, biostatistics, bioinformatics, clinical chemistry, pharmacology, nursing, bioethics, patient advocacy, and patient coordination. Interviews were conducted with 12 members of the MGTB from medical oncology, radiation oncology, medical genetics, pathology, bioinformatics, biostatistics, epidemiology, and patient coordination. Figure 1 provides a description of the MGTB participants by area of specialization and whether they participated in only the participant observation component of the study or both the participant observation and interview components. Interviews ranged in length from 25 to 49 min. Observational data is presented below in narrative form, and data drawn from the interviews is primarily presented in block quotes, though data presented on the practices of the MGTB were collected both through observations and interviews. Given the small sample size, and to protect MGTB members’ identities, we refer to interviewees as treating physicians, physician-scientists, and basic scientists.

The goal of the MGTB was to provide a forum for oncologists to present cases of breast cancer patients who had undergone FoundationOne™ testing^1^ to a multidisciplinary group of experts to aid in interpreting test results and making patient care recommendations. Twenty six individuals participated in the MGTB during its first year, though MGTB meetings typically involved five to 15 attendees, including at least one medical oncologist, a pathologist, a bioethicist, and the patient navigator.

For each case, the physician ordering FoundationOne™ testing presented the patient’s medical history, the pathologist showed and described the patient’s pathology slides, and a discussion of the patient’s FoundationOne™ test results followed. Results included individual genetic markers that may be related to the patient’s cancer, variants of unknown significance (VUS), and annotated explanations supporting the relationship between each mutation or genomic alteration and drug pathways. The MGTB discussed every marker reported by FoundationOne™ to make recommendations regarding: 1) FDA-approved therapies for breast cancer treatment; 2) therapies approved by the FDA for use in other tumor types; and 3) clinical trials enrolling patients with genomic markers identified in the patient’s tumor. The MGTB discussed treatment and research options and made recommendations for each genomic marker by consensus. The MGTB established levels of evidence for each recommendation based on Simon-Paik-Hayes biomarker guidelines [26]. The meeting moderator developed a web-based template to record, manage and report recommendations to ordering physicians. Over the course of the study, six treating physicians presented their patients’ cases to the MGTB, and the board’s recommendations became increasingly consistent and streamlined as participants became more familiar with the patterns of reported in FoundationOne™ reports. Systematizing the approach to reporting MGTB recommendations for ordering clinicians was an important and deliberate goal for establishing legitimacy, consistency and ethicality of the MGTB, and may have influenced the treatment recommendations of the MGTB in terms of ensuring consistent and thorough review of FoundationOne™ reports.

Genomic testing and presentation to the MGTB was left to physician discretion, however, all breast cancer patients’ cases sent by the hospital to Foundation Medicine for genomic testing during the study period were presented at the MGTB. Between October 2013 and November 2014, the Breast MGTB reviewed cases of 40 female patients with advanced metastatic breast cancer (Table 1). Ages ranged from 34 to 77 (median age 54). Tumor types in this population of breast cancer patients included 25 (63%) triple negative, 12 (30%) hormone receptor positive (ER or PR ≥1%), and three (7%) HER2 overexpressed (see Table 1). Patients reviewed by the Breast MGTB all had advanced disease, in distinction to the standard breast tumor board, where patients are almost exclusively presented at initial diagnosis so mostly have early stage disease.

**Table 1:** Description of tumor types reviewed by MGTB, *N* = 40

Tumor Type	Number	Percent
Triple negative	25	63
Hormone receptor positive/HER2 negative	12	30
HER2 overexpressed	3	7

A basic scientist explained: “These [MGTB] patients are the complicated ones. That’s why we’re sending them out for genomics because we’re looking for other options, ‘cause we’re otherwise out of options.”

A treating physician explained that indications for undergoing genomic testing included progression on standard breast cancer therapies (one for triple negative patients, two for hormone receptor positive patients), and lobular carcinomas with rare HER2 mutations with positive results. The preponderance of triple negative cases reviewed by the MGTB was explained by one treating physician in the following way:

“Unfortunately in that subgroup of women [triple negative patients], we still lack long-term sort of treatments that help them in metastatic setting. So I probably, after a couple of lines in metastatic setting for triple-negative breast cancer, I think I would do the FoundationOne™ testing for these patients.

This quote illustrates that the aggressive nature of these tumors and the few standard treatment options available to these patients makes FoundationOne™ testing an appealing option for the triple negative patient population.

FoundationOne™ reports included recommendations for on-label and off-label drug therapies and clinical trials that targeted genomic alterations in the patient’s tumor. The MGTB made recommendations based on actionable findings: mutations with associated therapies or clinical trials. The MGTB recommended referring most patients to clinical trials enrolling individuals with their genomic profile and clinical history (34, 85%) (see Table 2). Phase I, II and III clinical trials were recommended when available. The MGTB recommended FDA-approved therapies for breast cancer for 13 (33%) patients. The MGTB used FoundationOne™ results to recommend referring more than a third of the patients to medical genetics (15, 38%) for evaluation of suspected germline abnormalities associated with lifetime cancer risk. MGTB recommendations for additional testing were not always limited to recommendations presented in the FoundationOne™ report. The MGTB reviewed the recommendations issued in the commercial laboratory report, but made their own recommendations based on the expert advice of the MGTB participants. The group did not make recommendations for drugs or clinical trials beyond those suggested in the commercial laboratory report, but the MGTB occasionally made recommendations for additional testing beyond the scope of the FoundationOne™ recommendations, including androgen receptor testing (5, 12%) for triple-negative patients with a FoundationOne™ finding of a VUS suggesting an androgen receptor mutation and confirmatory HER2 testing for two patients (5%) for whom the patient’s chart, disease progression, and/or FoundationOne™ HER2 mutation statuses were incongruent. The MGTB did not recommend any off-label use of drug therapies approved by the FDA for use in other tumor types. The organizers of the Breast MGTB made a decision early on in establishing the group that if there was insufficient data for FDA approval of genomically-targeted agents for use in breast cancer that the MGTB ought not recommend it for breast cancer patients outside of a clinical trial context.

**Table 2:** Recommendations from MGTB, *N* = 40

Recommendation	Number of Patients	%	Number of Patients who followed MGTB recommendations	%
Genetics consultation	15	38	3	20
Clinical trial	34	85	6	18
FDA-approved therapies				
• On-label	13	33	5	38
• Off-label	0	0	0	0
Additional testing/biopsy				
• Androgen receptor testing	5	12	5	100
• Repeat HER2 testing	2	5	2	100

A physician scientist described the tenor of recommendations made by the MGTB positively: “It’s very good. I think Breast [MGTB] tends to be very conservative. After listening to just the general Genomic Tumor Board that we have had here, and listening to other Tumor Boards, non-genomic Tumor Boards, about … off-label use of different drugs in other clinical settings, I think there are groups that are a little bit more aggressive.”

MGTB participants were proud of the fact that they limited recommendations to standard lines of breast cancer therapy, clinical trials, and referrals to medical genetics. Treating physicians explained that the MGTB’s conservatism in relation to recommending off-label use of FDA-approved therapies reflected ethics of beneficence and nonmaleficence; they were uncomfortable recommending FDA-approved drugs that might harm breast cancer patients without proven benefit in their cancer type. The preponderance of recommendations from the MGTB that patients consider enrolling in targeted clinical trials reflected the lack of FDA-approved drugs for treating breast cancer tumors with specific genomic characteristics, and a recognition that participation in clinical research holds potential for different benefits, risks, and harms than standards lines of therapy.

### Benefits

All interviewees described advantages of establishing the MGTB, including providing more precise patient care recommendations and physician professional development. Interviewees highlighted the importance of teamwork in interpreting genomic data and making recommendations, and how this forum provided opportunities for interdisciplinary discourse. A treating physician noted:

“The participation has been spectacular… We have the cases presented and then have input from individuals from clinical medicine, from basic science, from bioinformatics and genomics about the testing …[T]he ethics perspective… is critical as we delve into realms that we don’t clearly understand, where we have limited information about certain findings and being able to…responsibly disclose that to the patient.”

Some physicians noted they would probably order fewer genomic tests if they did not have the MGTB’s interpretive support, and that the MGTB’s recommendations provided backing for the direction they were planning to suggest for their patients.

Another benefit mentioned was professional education through convening an MGTB:

“The ‘value added’ to the Genomic Tumor Board is the other thing that probably doesn’t get discussed too much is the overall ‘raising of the tides in the harbor,’ so to speak, ‘raises all ships.’ … it’s a great forum to discuss genomics and…serves as an educational forum, I think, for all physicians.”

Along these lines, some basic scientists noted feeling responsible for drawing attention to matters of scientific relevance in interpreting FoundationOne™ reports that may not capture the attention of oncologists. As one explained:

“Some [clinicians] just don’t have the time and they…don’t have probably even the training …to really understand genomics in that sense, and so until clinicians are trained in genomics … which I’m assuming will happen years going forward … this is really proving to me that you need a team of people to make this happen.”

### Challenges

We observed and participants identified technical and ethical challenges of incorporating genomic testing into patient care. For example, analysis of meeting and interview transcripts revealed that though inherently multidisciplinary, the MGTB’s discussions and recommendations were largely driven by medical oncologists with occasional input from basic scientists and physician-scientists. This dynamic remained constant during the 13 month time frame of the study. Though the rhetoric of inclusion was universal in participants’ descriptions of the MGTB, it was primarily treating physicians who conferred consensus of the group’s recommendations. Interviewees noted that expertise reflected in the recommendations was heavily influenced by who participated in MGTB meetings. Several called for increased substantive contributions from basic and translational scientists, pharmacologists, and medical geneticists to optimize the MGTB’s capacity, who were specifically valued for their expertise in tumor biology, interpreting genomic test results, referring patients to specific clinical trials, and their understanding of disrupted pathways that can predict response to FDA-approved treatments. However, a basic scientist noted that the format of the FoundationOne™ reports limited scientists’ ability to contribute to the MGTB: “[W]e can’t take advantage of expertise in bioinformatics because we’re not given any information on the bioinformatics.”

The absence of regular representation from medical genetics and genetic counseling was noted by several interviewees as a crucial challenge for the MGTB; a genetics professional participated in just one meeting during the thirteen month observation period. One basic scientist called for expanding the criteria for MGTB membership, advocating for the inclusion of nurses and social workers “because I think that patients will tell the nurses and social workers things that they will not tell the doctors. In fact I know they do.” However, the composition of the group remained constant during the study period.

Disciplinary differences were apparent in how MGTB members evaluated the quality of the FoundationOne™ test, yet participants’ concerns about the utility of the test were rarely mentioned in the context of the MGTB meetings. Treating physicians we interviewed focused on how genomic testing is: “just one of the tools we have in the toolbox. You don’t use every tool on every patient, and not every doctor uses every tool.” A physician-scientist explained: “In practice (and I don’t mean this just here), I think [precision medicine] it’s still a little ways away from really having a direct impact, I think a lot of mutations that are screened for on those [FoundationOne™ tests] are still not actionable clinically.” Though this study did not systematically collect data on how genomic test results informed ordering physicians’ recommendations for each patient’s care, treating physicians we interviewed conveyed that the clinical utility of genomic test results was low for many patients, and was significant in impacting treatment course for a few (see Table 2 for the number of patients whose course of treatment was informed by MGTB recommendations). As one treating physician explained:

When we get results that are useful, it can be very useful and beneficial to the patient. But more often than not, the results are of interest, potentially hypothesis-generating, and have little impact, little clinical impact, on the patient’s current state and seem to only benefit a small proportion of our patients.

Clinicians struggled with how to strike an appropriate balance of hope and realism regarding the actionability of genomic test results, raising questions about the role the MGTB should play in addressing the implications of their recommendations for patient care.

In light of these limitations, the MGTB’s discussions focused on what recommendations *could be made* with the knowledge of patients’ treatment histories and the incomplete insight provided by genomic testing. Some basic scientists noted that FoundationOne™ reports were insufficiently detailed or too incomplete to assess the value of these recommendations. While some were confident in the accuracy of reports, others expressed frustration that reports did not convey levels of confidence in each recommendation, particularly for germline variants. A physician-scientist also questioned how Foundation Medicine validates its product:

“They have teams of people to study the mutations, do literature searches, figure out eligibility for clinical trials, and so … we probably don’t rigorously question all they put in their report as often as…we should. You know, we pay them to do the test and assume that they’re doing it correctly, but…I think that more validation is what we need.”

The fact that the MGTB only reviewed patients with advanced disease complicated the process of making recommendations, as FoundationOne™ reports regularly identified FDA-approved therapies patients had already exhausted and clinical trials with exclusionary enrollment criteria. The tone of MGTB meetings suggested that patients were committed to aggressively treating their cancer, though occasionally a treating physician would mention limitations the patient or her family had put on the types of care they were willing to accept (e.g., chemotherapy via pill or infusion; aggressive vs. palliative care; work, family, or insurance coverage considerations) or willingness to travel to participate in a clinical trial. Nevertheless, in most cases the MGTB recommended shifting away from standard therapies toward clinical trials. FoundationOne™ reports clinical trials sites that are open nationally and internationally, and at the time of this study few were available in the city where patients were receiving care. The MGTB typically looked to ClinicalTrials.gov, the United States’ registry for public and private clinical studies involving human subjects, for genomically-targeted oncology clinical trials, but limited its recommendations to trials with open sites in the region, recognizing that patients may be most likely to consider participating in trials within driving distance.

Most interviewees mentioned they would like updates on patients reviewed by the MGTB, to learn how treating physicians and patients made use of recommendations, and patient outcome data on genomically-targeted therapies and clinical trials. A physician-scientist explained:

“I would like to see how many people were actually able to…be on a clinical trial…to evaluate whether or not if they were put on a different drug…it helped them or extended their life. I’d also like to know how they felt about the whole experience, whether or not they thought it was worthwhile, especially [because] it’s a lot of money.^2^ So they’ve spent a lot of money on these things and so it would be nice to know if they thought it was of benefit.”

Participants thought these data would help them assess the utility of FoundationOne™ and the MGTB’s recommendations for informing patient care, though this data was not reported back to the MGTB during the study period.

Finally, not all participants considered FoundationOne™ ethically neutral. Specifically, participants wondered if patients understood they might receive unexpected or unwanted information, as genomic tumor analysis might reveal germline mutations with implications for personal and familial lifetime health risks. MGTB participants worried that if patients knew that tumor analyses could illuminate germline risks it may perpetuate mistrust and misunderstanding of genetic testing. A physician-scientist also wondered if more explicit informed consent processes should be in place for sending prior tumor biopsies for genomic testing to protect patient autonomy. A treating physician stated:

“[There are] huge ethical issue[s] with many…social, legal and other implications because of the cost, the potential harm…I think we have to try and develop some sort of controls around the situation by setting up guidelines in… a logical way…for [the] community, for all of us, for the oncology world, because we’re all struggling with it and it’s here right now…so we have to catch up.”

This statement suggests that MGTB members saw themselves as trying to get in front of a deluge of genomic testing that is rapidly making its way into clinical oncology without requisite knowledge or procedures in place to ensure well-informed, genomically-guided patient care. As a treating physician noted:

“We need more structure around the way we’re putting together the recommendations, and the delivery of the recommendations, because right now it’s still very much… a work-in-progress where we’re trying to assess what the level of evidence is to even make a certain recommendation…but we have not yet delved into how…the treating oncologist actually delivers the information and what kind of advice they should be giving and exactly how we suggest that [information] be given.”

## Discussion and conclusion

This paper reports on a case study of the establishment of the first Breast MGTB. Findings suggest both feasibility and desirability of pooling professional expertise of clinicians and scientists in reviewing and making recommendations for genomically-guided breast cancer care. This study also suggests that there are technical and ethical challenges to institutionalizing a Breast MGTB.

This MGTB provides a unique forum to discuss genomic test results for cancer patients. While the MGTB resembles a regular multidisciplinary tumor board in its use of a standardized template to issue recommendations to treating physicians based on national guidelines for evidence-based breast cancer treatment [21, 22], it departs from a typical breast tumor board by involving non-clinicians in interpreting commercial test results for a single cancer type. A dynamic process unfolded in the MGTB’s first operating year in which the interpretation and standardization of recommendations continually evolved. This case study confirms the feasibility of standardizing interdisciplinary evaluation and recommendations. This was especially evident in the MGTB’s uptake of levels of evidence for making recommendations and reluctance to recommend FDA-approved therapies for off-label use, which is consistent with the dominant epistemological frameworks in oncology of adherence to national guidelines supporting evidence-based medicine and randomized controlled trials [22, 27, 28] and the bioethical principles of beneficence and nonmaleficence [29].

The Breast MGTB’s commitment to standardizing the integration of molecular tumor analyses into metastatic patient care signals what Nelson and colleagues characterize as a new sociotechnical regime in oncology focused on actionability of genomic results, “whereby the articulation of molecular hypotheses and experimental therapeutics become central to patient care,” through integration into existing clinical practices, decision-making, clinical trials, and health care infrastructures [30]. This case study confirms that molecular explanations inform the MGTB’s recommendations, but perhaps not yet to the extent of constituting an actionable regime. The MGTB primarily recommended referring patients to clinical trials, yet several participants cautioned that when patients had already exhausted standard therapies their expectations needed to be tempered when approaching genomic testing as an avenue for accessing new treatments or clinical trials for which they would be eligible. This suggests that FoundationOne™ testing is “actionable in principle” more than in practice in that many tumor mutations can be linked to FDA-approved therapies and/or clinical trials [31], but access to targeted drugs and clinical trials is still limited [15] and how MGTB recommendations impact patient outcomes is as of yet unknown. Hence, clinical utility is still largely promissory. This is evident in the levels of evidence the MGTB assigned to recommendations based on confidence in degree of actionability of results, and how the MGTB limits clinically actionable results to drug targets that are FDA-approved for breast cancer. The promissory tenor of genomic testing is reflected in our findings that treating physicians perceived the clinical utility of genomic testing to be significant for few patients but low for most patients, which was further evidenced through the low uptake of MGTB recommendations related to genomic testing by patients whose cases were reviewed. While these findings are consistent with previous research on follow up on tumor board recommendations [15, 19, 20], our study cannot explain the specific factors that contributed to uptake of MGTB recommendations by physicians and patients.

Another key finding included the widely-recognized importance of a multidisciplinary team, with experts in science, genomics, and ethics, which is aligned with the makeup of other genomic tumor boards [13]. Yet, treating physicians drove the Breast MGTB’s recommendations during its first year, which may reflect modeling the MGTB on a standard breast tumor board. To capitalize on the MGTB’s multidisciplinary expertise, deliberate efforts may be necessary to promote substantive contributions by clinicians and scientists unaccustomed to participating in tumor boards. As Parker and colleagues have also suggested [15], bolstering the attendance and engagement of professionals from medical genetics and pharmacology are especially warranted to increase substantive expertise and understanding of genetics and drug pathways. The potential for dependence on commercial laboratory interpretation of test results poses a problem for the rapid integration of un(der)-validated genomic tests, posing risks to the integrity of clinical judgment and patient care [5]. This may be especially important if the MGTB is to avoid overreliance on the commercial laboratory’s interpretation of genomic test results, a concern raised both by physician-scientists and basic scientists who noted the group’s relatively uncritical acceptance of the validity of (FoundationOne^TM^) results. Participants also noted benefits of increasing the participation of professionals who may be particularly useful for securing informed consent for genomic testing, managing incidental germline findings, and serving as educational resources for MGTB members [32].

Finally, all interviewees described benefits of convening the MGTB for patients and especially physicians, with one participant referencing the aphorism “a rising tide lifts all boats” to signify the communal benefits of pooling expertise to enhance genomic knowledge. While the stated goal of the MGTB was to improve patient care through targeted therapeutics, an underlying, and increasingly prominent, goal became educating clinicians about genomic markers, drug pathways, and the interpretation genomic test results. These goals do not negate one another, but occur in parallel, sometimes complementarily but with potential for conflict. This finding reflects the current state of genomic medicine, with its technical limitations and the unknown potential for impacting patient outcomes, but also raises the importance of acknowledging how the MGTB can be of use to physicians and patients considering testing. Of interest is how the MGTB can balance responsible stewardship of genomic technology in promoting cancer patients’ autonomous decision-making and professional education on incorporating genomics into clinical practice with a holistic understanding of the benefits and limitations of technology. Adequate informed consent poses a significant challenge to integrating genomic tools into oncology for two reasons. First, low genetic literacy can influence patients’ comprehension of the differential implications of germline and somatic testing [2]. Second, most oncologists have minimal training and confidence in genomic testing and interpretation of results [2, 33, 34]. Thus, in addition to promoting genomic expertise among oncologists through professional education [15], an MGTB is well-positioned to promote better informed consent through educating both physicians and their patients. Previous research has suggested that involving patients in multidisciplinary tumor board meetings deserves further consideration as an avenue for ensuring that tumor board recommendations reflect patient values [20]. This may be one pathway to both educate metastatic breast cancer patients about what genomic testing can realistically offer, and to assess how patient values can inform MGTB deliberations regarding recommendations for treatment.

This study has three primary limitations. Our analysis is limited to a single MGTB and reflects the dynamics at one institution; there may be local variability in the approaches and implementation of MGTBs. Furthermore, this study did not systematically track patient outcomes; a clinical report presenting information about patients presented at the MGTB will be published separately. Finally, this study did not gather the perspectives of patients who were offered FoundationOne™ testing. Patient viewpoints and the factors that contributed to their decisions to undergo testing and engage MGTB recommendations are unknown.

This case study presents several directions for future research, including assessment of professional variability in offering genomic testing in oncology and making treatment recommendations on the basis of genomic findings, and how physicians communicate the risks and benefits of genomic testing and respond to MGTB recommendations. Factors that influence patients’ decisions to undergo or forego genomic testing and their responsiveness to MGTB recommendations also warrant attention. Finally, this study raises questions about broader ethical and social implications of offering genomic testing to advanced breast cancer patients with the aim of improving treatment options, but more often than not, presenting opportunities to participate in clinical trials research. Engagement with these issues will provide a more holistic understanding of the ethical and social implications of the clinical integration of genomic technology in oncology.

## Appendix

### In-depth interview guide

Impressions of involvement in the MGTB

1. What is your professional role on the Multidisciplinary Genomic Tumor Board (MGTB)?
2. Now that the MGTB has been meeting for several months, what is your impression of your experience thus far?
  - What has been going well?
  - What areas require improvement?
  - How well represented are various disciplinary perspectives in MGTB deliberations?
    i. Are there particular perspectives that you have found especially helpful or interesting?
    ii. From which disciplines would you like to hear more?
3. What do you think of the presentations of cases and (FoundationOne^TM^) reports?
  - How useful is it to receive the (FoundationOne^TM^) reports before the meeting?
  - What do you think of the format of the MGTB meetings? (proceeding from clinical history of patient to (FoundationOne^TM^) report to action plan)?
4. What do you see as the benefits of convening the MGTB?
5. Conversely, what do you see as the drawbacks of having an MGTB analyze and make recommendations regarding patient care?
6. In the course of the MGTB meetings, the participants have also weighed in on (a commercial platform). What are your impressions of the process of developing (a commercial platform)? What impact do you think the platform will have on clinical care once implemented?

Determining Clinical Utility

7. Would you please describe how (FoundationOne^TM^) reports patient results?
  - In your opinion, what factors influence the MGTB‟s interpretation of (FoundationOne^TM^) reports?
  - In your opinion, are there any changes you would suggest for how patient results are reported?
8. How does the MGTB make recommendations regarding therapeutic options and clinical trials?
  - If it were up to you as an individual, would you have made the same recommendations that the MGTB has thus far? Why or why not?
  - In your opinion, how does clinical utility factor into the recommendations that are made?
  - What are the most important criteria for deciding that genomic data might have clinical utility? When you make a decision, what considerations are most important?
9. How would you describe the process for making a recommendation? What are your impressions?
  - Are there cases that have been presented that stood out to you as unique or noteworthy? What make them stand out to you? (e.g. straightforward, ambiguous)

Use of MGTB recommendations

10. Are you a clinician who orders (FoundationOne^TM^) tests for breast cancer patients?
  - If yes, how useful are the MGTB‟s recommendations regarding (FoundationOne^TM^) test results? How do you communicate (FoundationOne^TM^) results and MGTB recommendations to your patients? How receptive have patients been to recommendations?
  - If no, what would you like to know about how these recommendations are utilized by clinicians and how results are conveyed to and received by patients?
11. Are (FoundationOne^TM^) reports and corresponding MGTB recommendations making their way into the electronic medical record?
  - What do you see as the implications of including these results in the electronic medical record? Or, of leaving them out?

Going Forward

12. What role do you think the MGTB should have in the future?
  - How should it be organized?
  - Who should be involved?
  - Should it expand its scope beyond reviewing (FoundationOne^TM^) test results for breast cancer patients? Why or why not?
13. What do you see as the most pressing ethical, legal and social implications of sequencing tumors of breast cancer patients?
  - How, if at all, do you think that the MGTB can mediate these challenges in the future?

## Abbreviations

ER: Estrogen receptor
FDA: Food and Drug Administration
HER2: Human epidermal growth factor receptor 2
MTGB: Multidisciplinary Genomic Tumor Board
PR: Progesterone-receptor
VUS: Variants of unknown significance

## Footnotes

1 Described by the commercial vendor as “a fully informative genomic profile that helps physicians make treatment decisions for patients with cancer by identifying the molecular growth drivers of their cancers and helping oncologists match them with relevant targeted therapeutic options”[35]

2 (FoundationOne^TM^) cost $5800 at the time of the study [36]
